# Injuries to the Eye

**Published:** 1870-04

**Authors:** 


### INJURIES TO THE EYE.

Among the most prominent causes for blindness, are the various injuries to which the *cornea* is subjected. Sometimes these wounds are so severe as to cause immediate loss of the eye, as happens when a pointed instrument is thrust through it, or a sharp projectile, as a piece of steel, iron or stone, comes with rapid velocity against it. In such instances, the wounds are generally so large as to allow some of the humors to escape and the eye to collapse, when blindness at once ensues.

But, by far the greater portion of such injuries are simple in their character, and are only rendered dangerous through neglect or unwise manipulation. It is this form of the difficulty that we desire to guard our readers against, and to exhibit to them wherein lies the danger.

One class of sufferers, are those who receive particles of cinders or coal upon the surface of the cornea, while riding in a railway-car. Such people at once apply the handkerchief, glove or coatsleeve to the eye, rubbing vigorously, with the hope of removing the offending particle, much upon the same plan that a house-maid would adopt in removing stains from a brass kettle. This process imbeds the foreign particle deeply into the cornea, where it excites inflammation and if allowed to remain, suppuration of the parts. The proper way to remove such an offending substance from the eye, is not to rub it at all, but to seize the first opportunity, when the car is not in motion, to have some young person, with nimble fingers, evert the upper eye-lid, upon the under surface of which, the irritating particle will almost invariably, be found, and can be then readily removed by means of a clean handkerchief. But, if you fail to find it in this manner, *still avoid any rubbing;* keep the eye as quiet as the nature of the case will allow, and you will find that during the night, while you sleep, the cinder or coal will find its way into the corner of your eye, where it can be washed away when you perform your morning ablution.

The next class, liable to wounds of the cornea, are our artisans—those who work in our rolling-mills and machine shops, and particularly our stone cutters. These occupations subject the eye continually, to accidents, and it has always been a source of wonderment to us that men so employed, do not wear some sort of a contrivance over their eyes, similar to that used by their brethren in the work-shops of England. The article referred to, consists simply of plate glass, half an inch in thickness, of crystal clearness, and mounted in light frames. The glass being very thick and mounted in flexible frames, is capable of resisting the force of a pistol-bullet, while its form is such as not to interfere with vision, giving entire protection to the eye from external injury. Certainly, such accidents are sufficiently frequent to render the adoption of some means of protection, worthy of consideration. *

But, while these fatal injuries to the eye are transpiring, those of less severity are of daily occurrence. Particles of steel and iron are continually flying from the lathe and chisel of the machinist, imbedding themselves in the sub-

* The glasses referred to can be ordered of Collingwood & Strang, of this city, and should be worn by every man engaged in the chipping of iron or stone.

stance of the cornea, from which position the sufferer attempts their dislodgement, by means of pen-knife, stick, or needle, clumsily manipulated, by the hand of his fellow workman. In this operation, serious injury is liable to be done the cornea, by denuding it of its *conjunctiva* or covering membrane, exciting inflammation that is soon followed by ulceration. It is then, that the patient is beset with all manner of suggestions from his sympathizing friends. One will recommend a flax-seed poultice, another cold-tea leaves, and the third, an "alumn-curd." Still another, will relate how a shop-mate was instantly cured with "Thompson's-eye-water," or a solution of sugar-of-lead. In this manner, suggestions and advice will be freely offered, any of which, if followed up, would ruin a sound eye, to say nothing of a wounded one.

Any foreign body which is allowed to remain within the substance of the cornea, for two or three days, will certainly excite ulceration, which, if not properly combated, and the offending particle carefully removed, will leave a scar that will partly or entirely, obstruct vision. Now, the proper way to have a piece of iron, steel or stone removed from the cornea, is to apply immediately to the nearest surgeon, who, with a delicate, sharp-pointed instrument, wil dislodge the particle, by thrusting the instrument behind it, while your eye is directed to some object upon the wall in front of you. No pain will be experienced in its removal, if the patient is careful to keep his vision fixed steadily upon some object, while the surgeon is effecting the dislodgement ; otherwise, the task will be painful and difficult to accomplish. After the removal of the offending substance, the eye may be bathed in salt water—about two grains of table-salt to one ounce of rain-water. Should the eye be very painful, six drops of laudanum can be added to the solution, with benefit. Never use any of the advertised nostrums, under the name of "eye waters, about your eyes. Avoid them as you would blindness.

